# Optimum soil frost depth to alleviate climate change effects in cold region agriculture

**DOI:** 10.1038/srep44860

**Published:** 2017-03-21

**Authors:** Yosuke Yanai, Yukiyoshi Iwata, Tomoyoshi Hirota

**Affiliations:** 1 Hokkaido Agricultural Research Center, NARO, Memuro, Hokkaido, 082-0081, Japan; 2 Hokkaido Agricultural Research Center, NARO, Sapporo, Hokkaido 062-8555, Japan

## Abstract

On-farm soil frost control has been used for the management of volunteer potatoes (*Solanum tuberosum* L.), a serious weed problem caused by climate change, in northern Japan. Deep soil frost penetration is necessary for the effective eradication of unharvested small potato tubers; however, this process can delay soil thaw and increase soil wetting in spring, thereby delaying agricultural activity initiation and increasing nitrous oxide emissions from soil. Conversely, shallow soil frost development helps over-wintering of unharvested potato tubers and nitrate leaching from surface soil owing to the periodic infiltration of snowmelt water. In this study, we synthesised on-farm snow cover manipulation experiments to determine the optimum soil frost depth that can eradicate unharvested potato tubers without affecting agricultural activity initiation while minimising N pollution from agricultural soil. The optimum soil frost depth was estimated to be 0.28–0.33 m on the basis of the annual maximum soil frost depth. Soil frost control is a promising practice to alleviate climate change effects on agriculture in cold regions, which was initiated by local farmers and further promoted by national and local research institutes.

Anthropogenic climate change has become a major societal concern, and knowledge about effective adaptations to climate change are gradually accumulating in public and private sectors in agriculture worldwide^1^. For example, in the Tokachi region, the largest potato (*Solanum tuberosum* L.) production area in Japan, soil frost depth has decreased since the late 1980s because of climate change^2^. Shallow soil frost development was found to facilitate the overwintering of unharvested small potato tubers^3^. Therefore, volunteer potatoes have become a serious weed problem in the local rotation cropping system and in seed production^3^,^4^. To overcome this problem, local farmers in the Tokachi region began to remove snow cover during winter by using agricultural machineries such as tractors and bulldozers to enhance the freeze-kill of potato tubers. However, the lack of knowledge and experience occasionally hindered the control of the depth of soil frost penetration^4^. Subsequently, a national research institute in collaboration with some local research institutes developed a web-based decision support system that assisted in controlling the annual maximum soil frost depth (D_max_). This system involves (1) real-time visualisation of the daily predicted soil frost depth by using a mathematical model to determine soil temperature at an arbitrary depth under the snow cover^5^ and (2) an alerting service suggesting the recommended timing for conducting snow cover manipulation at each field. This system suggested that, for soil frost control, the D_max_ should range from 0.3 to 0.4 m in order to ensure the eradication of potatoes with minimised labour, cost, and risk in agriculture (see Fig. 1)^3^. Thus, controlling soil frost depth by using a scientifically sound method has enabled the management of volunteer potatoes over an area of several tens of hectares per farming household without requiring agrochemicals or heavy labour in summer^4^. At present, volunteer potato management by using the soil frost control method has become common among the farmers in and around the Tokachi region, eastern Hokkaido, northern Japan.

In addition to volunteer potato management, soil frost control is thought to have multiple effects on agriculture in cold regions^6^. For centuries, soil frost has been considered to have adverse effects on agriculture, such as soil erosion during the snow-melting period^7^,^8^ and delays in the initiation of agricultural activity because of the excessive wetting of soil in the early spring. Recently, there are concerns of temporarily large emissions of a greenhouse gas (nitrous oxide) immediately after snowmelt during soil thaw^9^,^10^,^11^,^12^. In addition, snowmelt water is known to immediately infiltrate the soil when the soil frost depth becomes shallow^13^,^14^,^15^, leading to an increased risk of water pollution by the leaching of residual nitrate in the surface soil^16^,^17^. These events caused by climate change suggest that a new optimum soil frost depth needs to be determined to avoid the negative effects on local agriculture and environment. In this study, we analysed the results obtained during field studies conducted in the Tokachi region to estimate an optimum soil frost depth. The suppression of volunteer potatoes was considered as the intended positive effect of the soil frost control, whereas smaller ratio of snowmelt water infiltration and increased nitrate loss from the surface soil were considered as the adverse effects. Because the majority (52%) of global potato production area is located between 44°N to 58°N^18^ and the Tokachi region is located around the southern boundary of the zone, our findings might help overcome the volunteer potato problem in the potato-producing cold regions, while avoiding the negative consequences.

## Results

The mean number of potato sprouts in spring (*λ*; sprouts ha^−1^) at the field plot (Table S1) was modelled by considering the number of potato tubers remaining after harvest in autumn (*Unharvested*; tubers ha^−1^) and the D_max_ (*D*_max_; m) as follows:


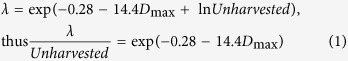


**Figure 2:**
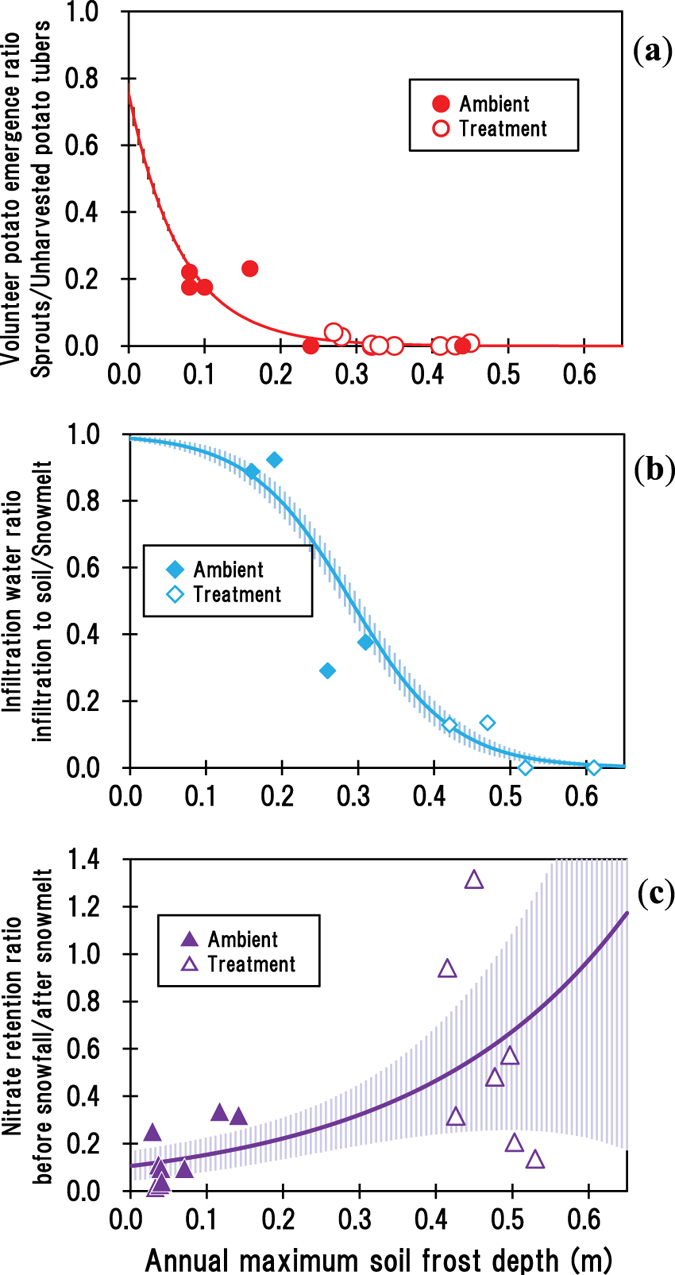
Parameterisations of the relationships between the annual maximum soil frost depth and(**a**) emergence ratio of volunteer potatoes, (**b**) snowmelt water infiltration ratio to soil, and (**c**) nitrate retention ratio at the surface soil. Solid line indicates the predicted value with 95% confidence interval. See also Supplementary Tables 1, 2, and 3.

The ratio of volunteer potato emergence defined as 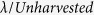 changed 0.24 times (=exp (−14.4 × 0.1)) by every 0.10 m increase in the *D*_max_ (Fig. 2a). Indeed, the emergence ratios of volunteer potatoes at the *D*_max_ of 0.20, 0.25, 0.30, and 0.35 m were 0.04, 0.02, 0.01, and <0.01, respectively. These results indicated that the current soil frost depth used (0.3–0.4 m) declines the emergence ratio to an unrecognisable level (<0.01).

The mean ratio (*q*; dimensionless) of snowmelt infiltration (*Inf*; mm) to the total amount of snowmelt water (*SnowMelt*; mm) at the experimental plot (Supplementary Table 2) was modelled by considering the D_max_ (*D*_max_; m):


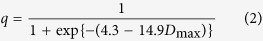


The mean infiltration ratios of snowmelt water to soil at the *D*_max_ of 0.10, 0.20, 0.30, 0.40, and 0.50 m were 0.95, 0.80, 0.48, 0.16, and 0.04, respectively, indicating a remarkable decline between 0.20 and 0.40 m of *D*_max_ (Fig. 2b). The *D*_max_ causing half of the infiltration ratio was around 0.29 to 0.30 m.

Nitrate retention to the surface soil because of deep soil frost development is expected to have similar dependency on the infiltration ratio of snowmelt water since it is highly soluble in water and would be transported in the soil solution. However, we found a remarkably different relationship^19^ (Fig. 2c). The mean nitrate content in the surface (0–0.4-m deep) soil after snowmelt (*μ*; kg N ha^−1^) was modelled by considering the D_max_ (*D*_max_; m) and nitrate content in the surface soil before snowfall (*AutumnN*; kg N ha^−1^) at the experimental plot (Table S3) as follows:


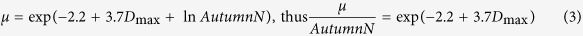


The nitrate retention ratio defined as 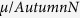 changed 1.45 times ( = exp (3.7 × 0.1)) by every 0.10 m increase in *D*_max_. Indeed, the mean (with 95% confidence interval) nitrate retention ratios at the *D*_max_ of 0.10, 0.20, 0.30, 0.40, and 0.50 m were 0.15 (0.08–0.23), 0.22 (0.13–0.31), 0.32 (0.19–0.44), 0.47 (0.24–0.69), and 0.67 (0.26–1.09), respectively. The *D*_max_ that caused half retention of nitrate at the surface soil was estimated to be 0.42 and deeper than 0.32 m when considering the mean and 95% confidence interval, respectively. This high uncertainty of soil nitrate retention ratio likely reflects the large spatial variability of the amount of snowmelt infiltration depending on the microtopography of the ground surface. In addition, measured and modelled nitrate retention ratio of over 1.0 can suggest the presence of nitrate discharge. Nitrification can be thought to occur after snowmelt where deep soil frost development easily releases decomposable organic matter such as cellular components of microbes^20^, because soil freezing does not significantly damage nitrifiers^21^. The measured soil nitrate profiles before snowfall and after snowmelt, as well as the time series of daily mean air temperature, snow cover thickness, and soil frost depth, are shown in Supplementary Figure 1.

**Figure 3:**
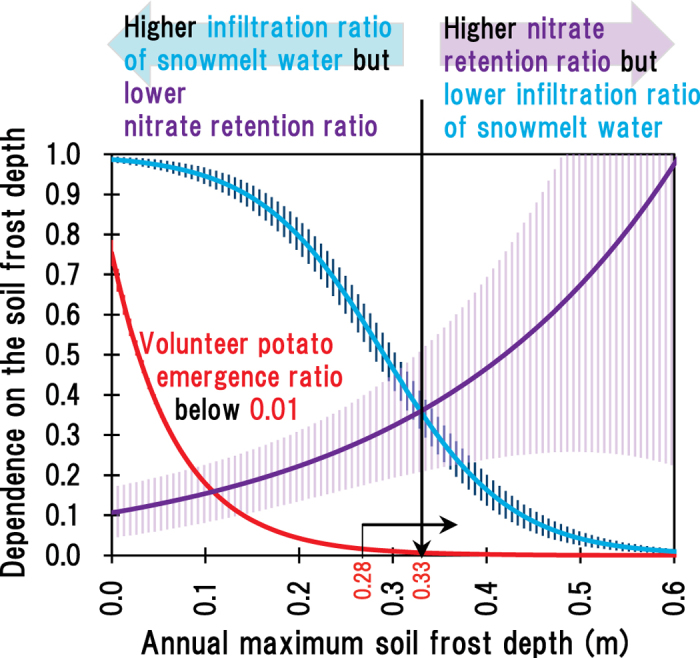
Derivation of the optimum soil frost depth (0.28–0.33 m) in relation to the ratio of volunteer potato emergence (red), snowmelt water infiltration (blue), and nitrate retention to the surface soil (purple). Solid lines indicate the predicted value with 95% confidence interval.

Considering the results of parameterisation on snow cover manipulation experiments as shown above and those obtained by agricultural/environmental implications, the optimum range of soil frost depth was suggested to be 0.28–0.33 m of D_max_ as follows (Fig. 3). The shallow limit of the optimum D_max_ (0.28 m) was relatively easy to determine by considering that it allowed the effective management of the emergence ratio of volunteer potatoes at around 0.01 or less. In this case, the infiltration ratio of snowmelt water was 0.53 and the nitrate retention ratio was 0.42. Conversely, another limit of optimum D_max_ (0.33 m) was difficult to determine reasonably because the nitrate retention ratio had large uncertainty in response to the D_max_. In addition, no exact thresholds were available to avoid flooding snowmelt water, soil erosion, and delaying agricultural activity initiation in response to the D_max_. Therefore, in the present study, we proposed the D_max_ limit to be simply the intersection point, i.e. 0.33 m (Fig. 3), indicating that the D_max_ balanced these two factors to avoid notable nitrate leaching. In this case, the infiltration ratio of snowmelt water was 0.35 and the nitrate retention was 0.51. Thus, we tentatively but quantitatively suggested the optimum range of the D_max_ as 0.28–0.33 m based on these considerations to eradicate volunteer potato emergence while managing nitrate leaching and snow-melt water infiltration. Further implications of the optimum D_max_ are discussed in the following section.

## Discussion

The proposed range of optimum D_max_ (0.28–0.33 m) was relatively narrower and shallower than the previously proposed value (0.3–0.4 m)^3^ owing to the additional considerations to the possible adverse effects of deep soil frost penetration on agriculture and environment. Higher nitrate retention ratio by deeper soil frost penetration can be an interesting trend, because soil frost control to manage volunteer potatoes could also allow the alleviation of groundwater pollution by nitrate. However, the D_max_ shallower than 0.35 m might be preferable to not enlarge the risk in temporarily increasing greenhouse gas (nitrous oxide) emissions from soil immediately after soil thaw^9^,^22^ (Supplementary Figure 2). Furthermore, importantly, the proposed D_max_ range was determined by our field data, which were mainly obtained at Andisol fields. Because Andisol is characterised by high permeability, the range of D_max_ might be similar to those in other fields having, for example, sandy soil. In contrast, soils having lower permeability might show smaller infiltration ratio of snowmelt water to soil even though the D_max_ is considerably shallower. The smaller infiltration ratio of snowmelt water to soil can allow higher retention ratio of nitrate at the surface soil layer. Therefore, the deeper limit of the optimum D_max_ might be less than 0.33 m as determined in our study (Fig. 3) in the case of soils having lower water permeability. In other words, our proposed optimum D_max_ might be applicable as a reference to achieve volunteer potato control with minimised adverse effects on the soil frost control for different soil types. If the D_max_ of 0.28 m is very deep for the fields to infiltrate surface-flooding snowmelt water, the target D_max_ should be modified based on the allowable level of volunteer potato emergence for individual farmers. Thus, maintaining the D_max_ within the optimum range might allow adaptation to and mitigation of climate change effects. For the extended application of soil frost control as the mitigation measure for climate change, further studies are needed to establish the relationships between seasonal dynamics of greenhouse gas (nitrous oxide) emission and soil management^23^.

The management of volunteer potatoes by using the soil frost control method is a promising strategy to adapt to climate change, which was initiated by some local farmers and supported by national and local research institutes, and it has become widely accepted by many local farmers and researchers. Soil freezing is known to be largely suppressed by thick snow cover, i.e. the D_max_ can be estimated using the freezing index (the summation of daily mean air temperatures for days with temperature of below 0 °C until the snow cover thickness becomes 0.20 m or more)^2^. Therefore, our on-farm soil frost control might likely be applicable in regions where air temperature drops sufficiently (mean air temperature, −12 to −5 °C during December to February) and the continuous snow cover appears early in winter when the mean precipitation reaches 50 to 150 mm during December to January^3^. Since the principle of soil frost control is to simply offset the heat-insulating effect of thick snow cover in order to expose soil surface to cold air, it can be easily performed over a large area of several tens of hectares by using common agricultural machineries by individual local farmers in a less time-consuming and labour-saving manner^3^. That is, the effectiveness of the soil frost control method has certain limitations depending on the magnitude and rate of climate change^24^. As mentioned above, if cold winter is accompanied by extremely short snowfall, the ambient D_max_ would be greater than 0.33 m; deep soil frost development would have adverse effects, because soil frost depth cannot be controlled without snowpack. In contrast, in the case of warm winter, soil freezing would be limited, and hence would not be sufficient to achieve the control of volunteer potatoes. However, the climate change scenarios for the Tokachi region^25^,^26^, i.e. mean air temperature in winter changes from current (−8 °C) to the late 21^st^ century (2081–2100; −5 °C), suggest that the D_max_ of around 0.3 m can be achieved^3^. Therefore, the optimum D_max_ determined in the present study could contribute to the improvement and provision of more options to perform multiple and immediate adaptation actions over different regions and times in potato-harvested bare fields. In addition to bare fields, the soil frost control method and concept of optimum D_max_ can be applied for the overwintering condition such as fields cultivated with winter wheat (*Triticum aestivum* L.)^27^. Furthermore, the soil frost control method can be utilised to prevent ground water pollution by facilitating soil nitrate remaining on the surface layer^16^ regardless of whether the fields were cultivated with potato or not. Scientists in the national and local research institutes need to provide scientific background for perspective practices of farmers in order to establish user- and environmental-friendly effective adaptation actions against climate changes in agriculture.

## Methods

### Statistical modelling

The optimum soil frost depth was estimated by re-analysing published data regarding the emergence ratio of volunteer potatoes and snowmelt water infiltration to soil; further, an on-farm snow cover manipulation experiment was conducted to determine nitrate retention at the surface soil owing to deep soil frost penetration. To characterise the responses to the annual maximum soil frost depth (D_max_), the generalised linear model (GLM) approach^28^ was applied.

### Volunteer potatoes

We assessed published data regarding the D_max_ (*D*_max_; m), numbers of unharvested potato tubers and emerged sprouts, and resulting emergence ratio of volunteer potatoes during the on-farm snow cover manipulation experiment conducted at 4 sites in the Tokachi region over 2 years (2010–11, 2011–12)^4^. For the statistical modelling, the reported numbers of remaining potato tubers after harvest and emergence of potato sprouts per unit square meter area (m^−2^) were multiplied by 10,000, and then rounded off to the closest whole number to convert to the unit (ha^−1^) and to an integer value (Table S1). In addition, the year of study and snow cover manipulation treatment at the same field were regarded as an independent study plot. Next, because a potato tuber has 0, 1, or several sprouts, i.e. there is no upper limit for the numbers in the emerged sprouts, we assumed that the observed variation in the potato sprouts per hectare (*Sprouting*) follows the Poisson distribution of mean *λ* (eq. 4). We set *D*_max_ as the explanatory variable, and the number of unharvested potato tubers per hectare (*Unharvested*) as the offset term in the linear predictor. Subsequently, the log link function was applied (eq. 5). The coefficients in eq. 5 (*β*_*s*0_, *β*_*s*1_) were estimated using the ‘glm’ function of the R software package^29^.


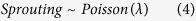



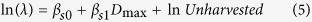


### Snowmelt water infiltration

We re-assessed published data on the relationship among the D_max_ (*D*_max_; m), amount of infiltrated water to soil, and amount of snowmelt water during the snow cover manipulation experiment at the Memuro Research Station (Hokkaido Agricultural Research Center, NARO: 143 °05′E, 42 °53′N) in the Tokachi region for over 4 years (2005–06^30^, 2006–07^30^, 2007–08^30^, and 2008–09^15^). In these studies, soil frost depth was controlled by removing the snow cover in early winter. The cumulative daily downward water flux at 0.5-m soil depth during the snowmelt period was set to the amount of infiltrated water to soil (*Inf*; mm). Similarly, the cumulative decrease in snow-water equivalent during the snowmelt period was set to the amount of snowmelt water (*SnowMelt*; mm). These values were slightly different from the published data^15^,^30^ because they were rounded off to the closest whole number to convert to an integer value for statistical modelling (Table S2). In addition, the year of study was regarded as an independent study plot. Based on the fact that the amount of infiltrated water to soil was no more than that of snowmelt water, i.e. data *Inf*/*SnowMelt* were between 0 to 1, we assumed that the observed variations in the *Inf* follows the binomial distribution of mean *q* and of the upper limit *SnowMelt* (eq. 6). We set *D*_max_ as the explanatory variable in the linear predictor and then applied the logit link function (eq. 7). The coefficients (*β*_*q*0_, *β*_*q*1_) in eq. 7 were estimated using the ‘glm’ function of R software package^29^.


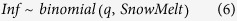



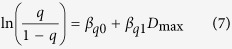


### Nitrate retention to the surface soil

We conducted an on-farm experiment to evaluate the nitrate retention in the surface soil after snowmelt at fields with different annual maximum soil frost depths (*D*_max_; m). The snow cover manipulation experiment was conducted at two experimental fields at the Memuro Research Station. The soil type is classified as Andisols derived from volcanic ash, which is the major soil type in the Tokachi region. Soil nitrate content at 0–0.40 m depth (kg N ha^−1^) before snowfall and after snowmelt was set to *AutumnN* and *Spring N*, respectively (Table S3). For the statistical modelling, the year of study and experimental field examined were regarded as an independent study plot. Next, because nitrate content is a continuous value and should not have negative values, we assumed that the variations in the observed nitrate content in the surface soil after snowmelt follows the Gamma distribution of shape parameter *s* and rate parameter *r* (eq. 8), which are related to the mean *μ* with *s*/*r* and the variance with *s*/*r*^2^. According to the definition of the dispersion parameter ϕ, these parameters are related to the variance with *μ*^2^ϕ; thus, *s* is 1/ϕ and *r* is 1/ϕ*μ*. We set *D*_max_ as the explanatory variable and *AutumnN* as the offset term in the linear predictor. Subsequently, the log link function was applied to the linear predictor (eq. 9). The parameters in eq. 8 (*s, r*) and the coefficients in eq. 9 (β_*N*1_, β_*N*2_) were estimated using the ‘glm’ function of R software package^29^.


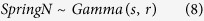



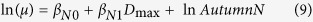


### Experiment in the winter wheat field

In 2008 and 2009, winter wheat (*Triticum aestivum* L.) was cultivated in an experimental field where the effect of tillage intensity on crop productivity and greenhouse gas fluxes of soil were tested^9^. Basal nitrogen fertilizer was applied at 60 kg N ha^−1^ following local conventional agricultural practices. Snow cover thickness was not manipulated during December 2008 to March 2009, i.e. no snow cover manipulation treatment was performed at the conventional tillage (CT) and reduced tillage (RT) plots, which have 8-m width from east to west and 48-m length from north to south (both were 384 m^2^ in size). These plots were 13 m apart from each other from east (RT) to west (CT). The soil frost depth was measured once weekly or more by using the frost tube method^31^, and the annual maximum soil frost depth (D_max_) was found to be 0.12 m and 0.14 m at the CT and RT plots, respectively (Table S3). In the succeeding year, 2009–2010, winter wheat was cultivated in a similar manner, and a snow cover manipulation experiment was conducted alternately for 3 snow cover compaction and ambient control plots; each experimental plot had a size of 64 m^2^. Snow cover compaction was performed twice a month in December 2009 and January 2010 by using a tractor^27^; the mean D_max_ of triplicated plots was 0.49 m and 0.47 m at the treatment plots compared to 0.05 m and 0.04 m at the control plots of CT and RT, respectively (Table S3). Soil samples were collected from each plot from 0–0.40 m by using a hand-auger at 0.10-m intervals in mid-December and early May to estimate the nitrate content in the surface soil before snowfall and after snowmelt. Well-mixed fresh soil samples were shaken with 2 *M* of potassium chloride solution for 60 min to extract nitrate, and its concentration in the extract was determined using the copper-cadmium reduction method by using a flow analysis system (QuAAtro; SEAL Analytical Gmbh, Norderstedt, Germany). The mean soil nitrate content (mg N kg^−1^ dry soil) for the 0.10-m thick soil was converted to the unit of kg N ha^−1^ by separately determining the layer-dependent bulk density, which was 0.77, 0.88, and 1.23 g cm^−3^ for the 0–0.10, 0.10–0.30, and 0.30–0.40 m depths of the CT plot, respectively, compared to 0.76, 0.77, and 0.58 g cm^−3^ for the 0–0.10, 0.10–0.20, and 0.20–0.40 m depths of the RT plot, respectively.

### Experiment in the corn field

In the experimental field located at the east of the RT plot, corn (*Zea mays* L.) was cultivated in summer in 2009 following the local conventional practice. After harvest in late August, 50 kg N ha^−1^ of ammonium sulphate was applied using a broadcaster to enhance corn residue decomposition. This field (approximate size, 1 ha) was maintained bare during winter and divided into two parts for a treatment (snow cover manipulation) and a control (ambient snow cover) plot. The snow cover compaction treatment was conducted twice a month during December 2009 and January 2010. The mean D_max_ in the control and treatment plots was 0.03 m and 0.42 m, respectively. Soil core samples were collected from each plot from 0–1.0 m depth by using an engine-auger in late November and early April to estimate the nitrate content in the surface soil before snowfall and after snowmelt. The core sample was cut in 0.10 m-intervals in the field, and a portion of fresh soil samples was shaken with 2 *M* of potassium chloride solution for 60 min to extract nitrate. The concentration of nitrate in the extract was determined, and the mean soil nitrate content (mg N kg^−1^ dry soil) for the 0.10-m thick soil was converted to the unit of kg N ha^−1^ as described above. The bulk density of the control and treatment plots was assumed to be the same as 0.83, 1.01, 0.95, 0.67, 0.91, 1.15, 1.12, and 1.10 g cm^−3^ for the 0–0.1, 0.1–0.2, 0.2–0.4, 0.4–0.5, 0.5–0.6, 0.6–0.7, 0.7–0.8, and 0.8–1.0 m depths, respectively.

## Additional Information

**How to cite this article:** Yanai, Y. *et al*. Optimum soil frost depth to alleviate climate change effects in cold region agriculture. *Sci. Rep.* **7**, 44860; doi: 10.1038/srep44860 (2017).

**Publisher's note:** Springer Nature remains neutral with regard to jurisdictional claims in published maps and institutional affiliations.

## Supplementary Material

Supplementary Material

## Figures and Tables

**Figure 1:**
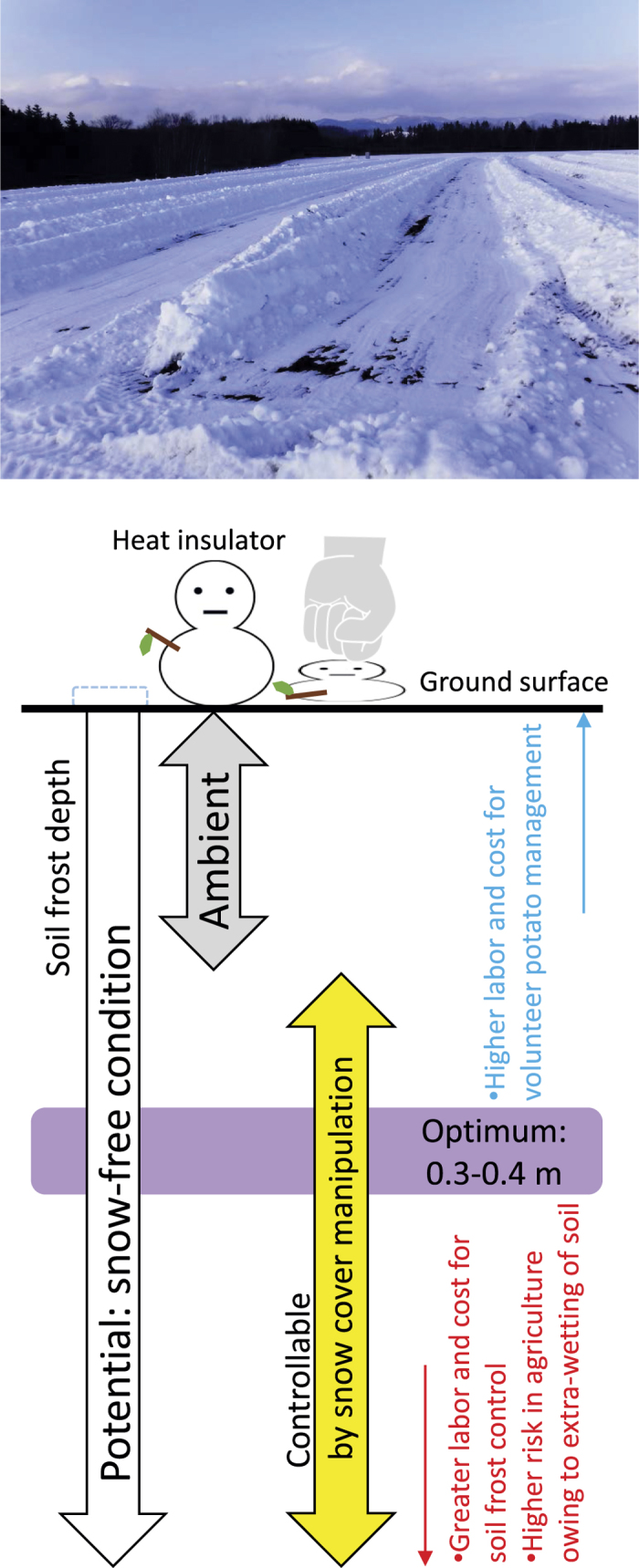
Outline of the ‘on-farm soil frost control’ technique and a conceptual diagram showing the optimum soil frost depth for the management of volunteer potatoes.
