# Supplementary material for: Optimum soil frost depth to alleviate climate change effects in cold region agriculture
Source: Sci Rep. 2017 Mar 21;7:44860. doi: 10.1038/srep44860 (PMC5359591; doi:10.1038/srep44860)
Supplement: Supplementary Material [file srep44860-s1.pdf]

Supplementary material

Optimum soil frost depth to alleviate climate change effects in cold region agriculture

Yosuke Yanai, Yukiyoshi Iwata, and Tomoyoshi Hirota

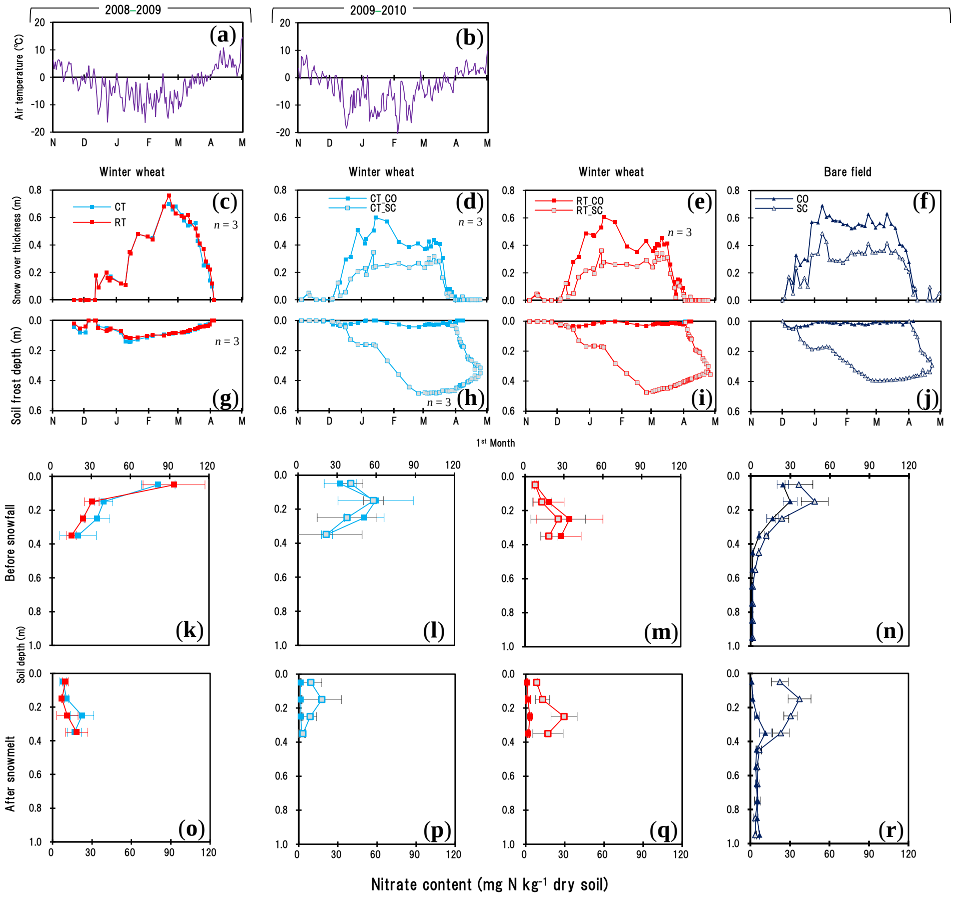

Fig. S1 On-farm snow cover manipulation experiment to determine the effect of soil frost depth on nitrate concentration profile in the soil at two experimental fields in the Memuro Research Station of NARO Hokkaido Agricultural Research Center.

CT: field managed with conventional tillage; RT: field managed with reduced tillage
CO: control with ambient snow cover thickness; SC: treatment for snow cover compaction
(**a**), (**b**): time series of the daily mean air temperature at the Memuro Research Station
Values shown in (**c**), (**f**), (**g**), and (**j**) are the mean of intra-plot measurements (*n* = 3), whereas those in (**d**), (**e**), (**h**), and (**i**) are the mean of inter-plot measurements (*n* = 3)
Values shown in (**k**) and (**o**) are the mean and standard deviation of intra-plot measurements (*n* = 3)
Values shown in (**l**), (**m**), (**p**), and (**q**) are the mean and standard deviation of inter-plot measurements (*n* = 3)
Values shown in (**n**) and (**r**) are the mean and standard deviation of intra-plot measurements (*n* = 1–3 and 1–6, respectively).

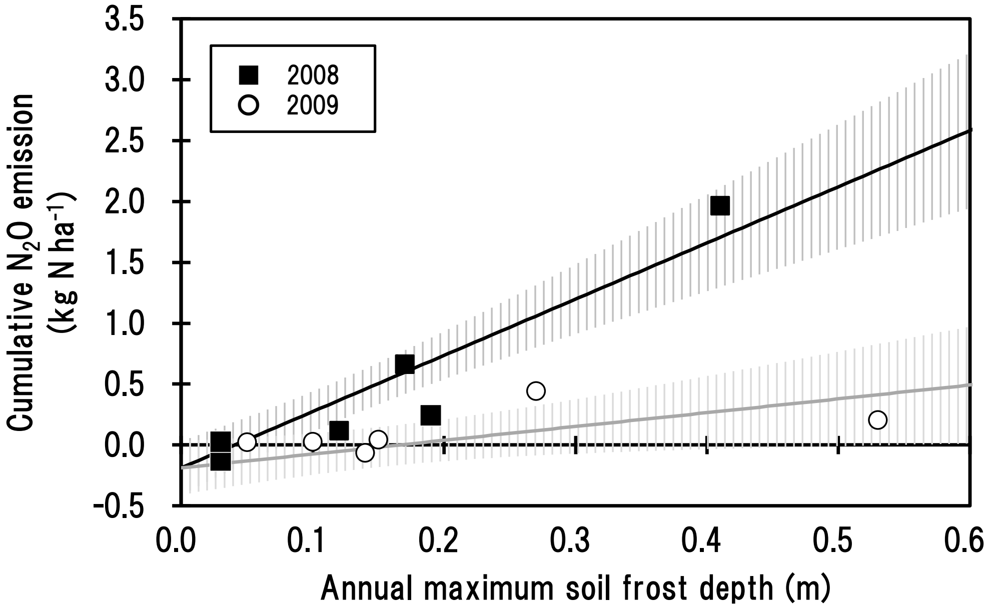

Fig S2. Relationships between the annual maximum soil frost depth and cumulative nitrous oxide emission from soil determined during the snow cover manipulation experiment[22]. Solid lines indicate predicted values by linear regression with 95% confidence interval. Years 2008 and 2009 indicate the cumulative period for nitrous oxide emissions (from November to April) of 2008–2009 and 2009–2010, respectively.

Supplementary Table 1. The soil frost depth, numbers of unharvested potato tubers, and emerged sprouts and emergence ratio of volunteer potatoes at the on-farm snow cover manipulation experiment conducted at 4 sites in the Tokachi region over 2 years.

Year	Site	Treatment	Observed D_{max}	Number of unharvested tubers	Number of emerged sprouts	Emergence ratio of volunteer potatoes
			m	tubers ha^{-1}	sprouts ha^{-1}	-
			D_{max}	*Unharvested*	*Sprouting*	*Sprouting/Unharvested*
2010–11	Shintoku	CO	0.16	59900	13860	0.23
2010–11	Shihoro	CO	0.44	58500	0	0.00
2010–11	Sarabetsu	CO	0.08	47300	8280	0.18
2011–12	Shintoku	CO	0.10	163400	28600	0.18
2011–12	Shihoro	CO	0.24	131900	0	0.00
2011–12	Sarabetsu	CO	0.08	115400	25400	0.22
2010–11	Shintoku	SFC	0.28	59900	1640	0.03
2010–11	Sarabetsu	SFC	0.45	47300	380	0.01
2010–11	Taiki	SFC	0.32	76200	0	0.00
2011–12	Shintoku	SFC	0.32	163400	700	0.00
2011–12	Shihoro	SFC	0.41	131900	0	0.00
2011–12	Sarabetsu	SFC	0.43	115400	30	0.00
2011–12	Taiki	SFC	0.35	184500	0	0.00
2010–11	Shintoku	SFCF	0.27	59900	2440	0.04
2010–11	Taiki	SFCF	0.33	76200	40	0.00

CO: ambient snow cover condition; SFC: soil frost control based on soil temperature model; SFCF: soil frost control voluntarily by farmers

D_{max}: annual maximum soil frost depth

Supplementary Table 2. The soil frost depth, amount of water infiltrated to soil, amount of snowmelt water, and estimated snowmelt water infiltration ratio in the snow cover manipulation experiment at the Memuro Research Station (NARO Hokkaido Agricultural Research Center) in the Tokachi region conducted over 4 years.

Year	Plot	Observed D_{max}	Infiltrated water to soil	Snowmelt water	Observed snowmelt water infiltration ratio
		m	mm	mm	-
		D_{max}	*Inf*	*SnowMelt*	*Inf/SnowMelt*
2005–06†	CO	0.19	83	90	0.92
	SR	0.47	15	111	0.14
2006–07†	CO	0.31	53	141	0.38
	SR	0.52	0	49	0.00
2007–08†	CO	0.26	10	34	0.29
	SR	0.61	0	9	0.00
2008–09‡	CO	0.16	183	206	0.89
	SR	0.42	19	150	0.13

CO: ambient snow cover condition; SR: soil frost control by snow cover removal

D_{max}: annual maximum soil frost depth

Adopted from †: Iwata et al. (2011)[28]; ‡: Iwata et al. (2011)[15]

Supplementary Table 3. Snow cover manipulation (compaction) experiment at the Memuro Research Station (NARO Hokkaido Agricultural Research Center) conducted to determine the effect of soil frost depth on the retention of nitrate in the surface soil layer (0–0.4 m depth) after the snowmelt.

Overwintering situation	Year	Plot	Loc	Observed D_{max}	Nitrate content at surface soil		Nitrate retention ratio
				m	kg N ha^{-1}		-
				D_{max}	*AutumnN*	*SpringN*	*SpringN/AutumnN*
Bare†	2009–10	CO		0.03	77	19	0.25
		SC		0.42	121	114	0.94
Winter wheat	2008–09	CT_CO		0.12	157	53	0.34
		RT_CO		0.14	144	46	0.32
	2009–10	CT_CO	N	0.03	181	3	0.02
			C	0.04	200	6	0.03
			S	0.07	104	10	0.10
		CT_SC	N	0.43	206	65	0.32
			C	0.50	123	25	0.21
			S	0.53	136	19	0.14
		RT_CO	N	0.04	90	10	0.11
			C	0.04	79	8	0.10
			S	0.04	165	7	0.04
		RT_SC	N	0.45	53	70	1.32
			C	0.48	111	54	0.48
			S	0.50	141	81	0.57

CO: ambient snow cover condition; SC: soil frost control by snow cover compaction

CT: conventional tillage; RT: reduced-tillage

D_{max}: annual maximum soil frost depth

Loc: Location in the plot. N: northern; S: southern; C: central between N and S

†: After corn cultication
